# Impact of Mask Type on the Effectiveness of and Adherence to Unattended Home-Based CPAP Titration

**DOI:** 10.1155/2019/4592462

**Published:** 2019-03-25

**Authors:** Magalí Blanco, Glenda Ernst, Alejandro Salvado, Eduardo Borsini

**Affiliations:** ^1^Sleep and Ventilation Unit, Hospital Británico de Buenos Aires, Argentina; ^2^Center for Respiratory Medicine, Hospital Británico de Buenos Aires, Argentina

## Abstract

**Objectives:**

To compare interfaces performance during home-based automatic titration (APAP).

**Methods:**

Retrospective study based on APAP titration from Obstructive Sleep Apnea Syndrome (OSA) patients.

**Results:**

707 patients, 513 men (70.6%), were titrated. Masks were 104 pillows (14.7%), group I (GI); 532 nasal (75.2%), group II (GII); and 71 oronasal masks (10%), group III (GIII). We found differences in effective pressure to the device (P90/P95) (GI: 7.13±1.9 vs. GII: 8.3±2.1 vs. GIII: 9.3±2.6 cmH_2_O, p <0.001) but not in final pressure titrated manually (GI: 7.9±1.4 vs. GII: 8.6±1.6 vs. GIII: 9.2±1.9 cm of H_2_O, p >0.5), where lower residual AHI for pillows was p <0.001 and leaks for nasal were p <0.001. No differences were found in compliance (hours) (GI: 6.3±1.2 vs. GII: 6.2±1.1 vs. GIII: 6.1±1.0, p <0.4).

**Conclusion:**

During auto-adjusting titration by CPAP-naïve patients, nasal masks had lower leak rates and nasal pillows presented a similar performance.

## 1. Introduction

Continuous positive airway pressure (CPAP) is the treatment of choice for patients with moderate to severe obstructive sleep apnea hypopnea syndrome (OSA). CPAP may be administered with a nasal interface, an oronasal mask, or nasal pillows [1, 2]. Even though CPAP therapy is known to be effective and long-term adherence has been consistent and repeated in clinical trials, reported adherence ranges from 40 to 80%. However, 10-20% of patients discontinue CPAP therapy after the first night of use due to discomfort from mask-related side effects [3–5].

It has been reported that half of CPAP patients experience mask-related adverse effects, such as leaks, pain, pressure ulcers on the bridge of the nose, nasal congestion, and a dry mouth or nose [5–8]. The type of interface has been found to exert an influence on therapy tolerance and adherence. Several studies have shown that oronasal masks have been associated with higher residual apnea-hypopnea indexes (rAHI) and higher pressures than nasal masks when used by the same patient [9–14].

With the purpose of reducing the occurrence of undesirable events, manufacturers have designed and marketed new masks. Among them are nasal pillows with two nostril inserts. Even though nasal masks are considered as the first-line interface to treat sleep obstructive disorders, some studies have shown that nasal pillows could be equally effective [7, 8, 15].

Auto-adjusting or automatic positive airway pressure devices use pressure-adjusting algorithms to detect respiratory events. Healthcare centers with a heavy demand for sleep tests and long waiting lists have suggested APAP as a nonconventional titration method. Auto-adjusting devices were not originally designed for this purpose. However, their use has spread because their internal memory can record working pressure variations derived from airway obstruction events throughout the night. These recordings enable analysis of such variable according to each individual patient.

APAP titration can be used at home for several nights, collecting a more extensive range of data. This approach allows physicians to assess night-to-night variability, shortens waiting lists, and lowers the operating costs related to conventional titration [16–20].

According to the last recommendations issued by* the American Academy of Sleep Medicine* (AASM), APAP titration is a valid option for patients with no significant comorbidities, provided that data are analyzed manually by experienced personnel [21] to titrate pressure at the sleep laboratory or at patients' homes using a self-administered approach without additional monitoring [21, 22].

The performance of masks and their impact on unmonitored titration processes have not been deeply studied. The objective of this study is to compare different interfaces in terms of short-term adherence and effectiveness in patients with obstructive sleep apnea during unattended home-based titration

## 2. Materials and Methods

### 2.1. Study Design

This is a retrospective and observational study conducted at Hospital Británico in the city of Buenos Aires, Argentina. The protocol was approved by the institutional review board and ethics committee (CRIHB number: #844).

### 2.2. Population

This is a retrospective study based on home APAP titrations systematically collected between October 2015 and December 2017 (26 months) from OSA patients referred to the Sleep Unit for a demonstration on the use of interfaces in an unmonitored setting. Researchers only included recordings from CPAP-naïve patients. Authors were not involved in the indication for CPAP therapy, which was prescribed by each patient's treating pulmonologist.

Patients diagnosed with obesity-hypoventilation syndrome, periodic breathing, or central apnea at baseline and those who needed other types of treatment (i.e., ventilation with two pressure levels, servo-controlled ventilation, and concomitant oxygen) were excluded from the study.

Baseline apnea-hypopnea index (AHI) in events/hour was obtained from PSG or RP recordings. Sleep scoring and standard definition according to severity of apnea and hypopnea syndrome was defined according to the traditional criteria of the American Academy of Sleep Medicine. OSA was classified in mild: IAH 5-14.9, moderate: IAH 15 to 30, and severe: > 30 events/hour [23, 24].

Anthropometric data (i.e., body mass index (BMI) and neck circumference (NC) were measured before delivering CPAP machines.

### 2.3. Mask Selection

Mask type, size, and model were selected according to our Unit's standardized procedure after a demonstration of the different interfaces performed by a physical therapist trained in sleep medicine. CPAP devices included either a standard nasal mask (an interface that covers the nose but not the mouth) or nasal pillows (mask with nasal inserts). Patients who did not tolerate smaller masks or who preferred larger masks were shown different models of oronasal masks.

After said demonstration, CPAP devices were turned on and tested for a few minutes with masks on. Patients were asked about comfort level and, if necessary, adjustments were made for greater tolerance and proper fitting. Finally, patients were asked to choose a mask.

Final mask selection depended on patient's preferences and the result of a 20-minute leak test of the APAP device. Patients and their spouses or roommates received basic training on the use of CPAP devices and masks. In addition, they were invited to take part in the patient education program offered at our Sleep Unit called “School of CPAP” [25].

### 2.4. Home-Based Unattended Auto-Adjusting CPAP Titration

We used auto-adjusting System One, Dream Station (Philips-Respironics; USA), S9 and Air Sense (ResMed-Sydney; Australia) devices. Unlike conventional CPAP, which uses fixed continuous positive airway pressure, APAP algorithms automatically adjust pressure through a flow sensor and adjust working pressure in response to respiratory events (apneas, hypopneas, limited inspiration, and snoring) until a normal respiratory pattern resumes. Minimum pressure was set at 4 cm of H_2_O and maximum pressure at 15 cm of H_2_O. Ramp features and expiratory pressure relief were not used.

Titration periods were consistent with effective Sleep Unit procedures (from 3 to 7 days) depending on the availability of appointments and devices. All nights with CPAP utilization in each patient were analyzed. When there were periods of no use or excessive leakage (above the compensation limit of the device), the night was not considered for the analysis.

**Figure 1:**
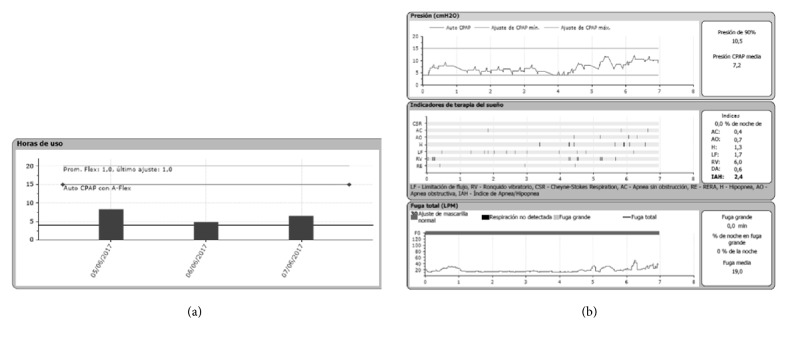
CPAP data during three nights (Software Encore Pro II). Note the vertical bars for use > 4 hours (a) and the pressure and leakage curves (b). The record corresponding to the night displayed showed no leakage and corrected rAHI with a pressure of 10 cm H_2_O (b).

**Figure 2:**
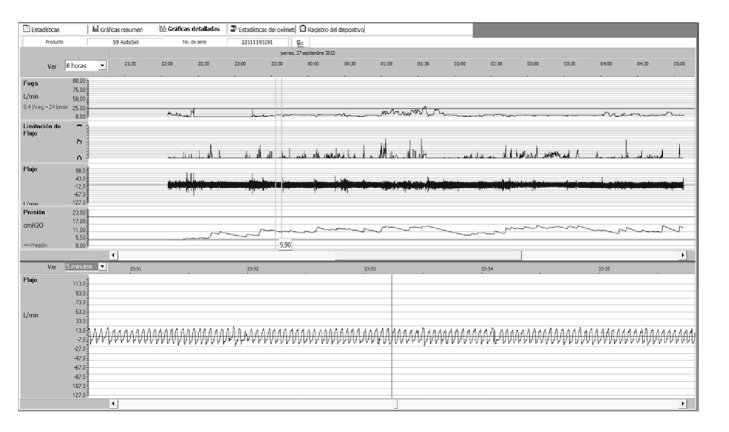
One-night CPAP data using ResScan Software. The pressure, flow, and leak channels can be appreciated (limit of leakage compensation up to 25 liters/minute). The effective titration pressure by visual analysis was established in 10 cm H_2_O.

Titration data were obtained after downloading the information from the CPAP machine SD card using Encore Pro II Philips-Respironics and ResScan-ResMed or ResMed Air View online platform for remote monitoring (Figures 1 and 2).

Effective pressure data were obtained after visual analysis of pressure/time curves by physicians and physical therapists trained in sleep medicine (Figure 2). Researchers analyzed > 20.000 hours of recordings of APAP use and obtained data on compliance, mean leak, and residual AHI. Minimum adherence was defined as using CPAP effectively at least for 4 hours/night during each study night. Optimum titration was defined as a rAHI of < 5 events per hour (events/hour). Titration was deemed acceptable when rAHI ranged between 5 and 10 events/hour.

Researchers compared APAP recordings according to interface type.

### 2.5. Statistical Analysis

Categorical variables are expressed as percentages and numerical variables as mean and standard deviation (±). Differences were compared using Fisher's test, Mann–Whitney test, or *χ*2. Titration pressures were compared using linear regression analysis. Statistical analyses were conducted using Graph Pad Prism-7.04™ software.

## 3. Results

707 patients were titrated: 513 men (70.6%) and 194 women.  Table 1 summarizes patient's characteristics.

According to the AHI per hour of sleep on polysomnography (PSG) or recording time on respiratory polygraphy (RP), patients were classified as having mild (4.5%) OSA (baseline AHI (mean) of 10.2 ± 3.3 events/hour), moderate (36.5%) OSA (AHI of 22.2 ± 4.1), and severe (59%) OSA (AHI of 47.7 ± 16.4). The mean titration period was 3.2 ± 0.8 nights.

**Figure 3:**
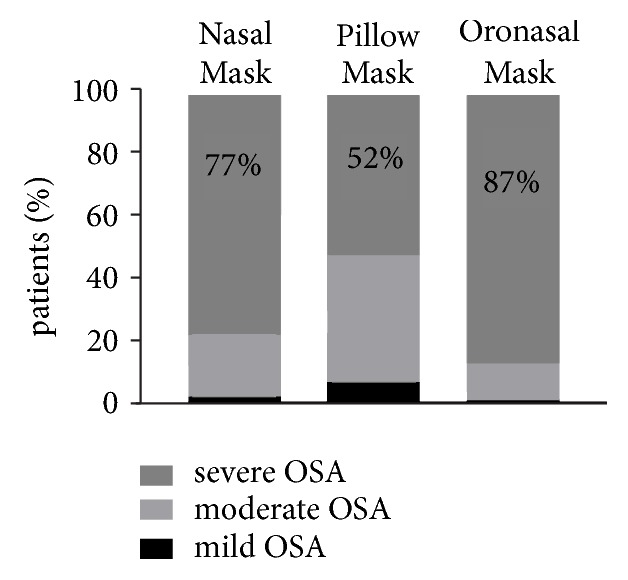
Type of interface selected according to AHI-based OSA severity.

The study assessed 3 types of masks categorized into 3 groups: 104 nasal pillows (14.7%) in group I (GI); 532 nasal masks (75.2%) in group II (GII); and 71 oronasal masks (10%) in group III (GIII). Group I had a larger proportion of moderate OSA patients, while group III mostly consisted of patients with severe OSA (Figure 3).

Compliance was similar across groups (6.2 ± 1.1 vs. 6.3 ± 1.2 vs. 6.1 ± 1.1 hours/night)* p* > 0.5. There were no differences between groups in terms of age (57 ± 12 vs. 59.4 ± 12 vs. 59.1 ± 12.2 years), neck circumference (41.4 ± 2.3 vs. 43.4 ± 4.1 vs. 43.7 ± 3.3 cm), body mass index (33.1 ± 6.6 vs. 34.2 ± 6.8 vs. 34.1 ± 6.4 kg/m^2^), and baseline Epworth Sleepiness Scale ESS (9.8 ± 5.7 vs. 9.8 ± 5.8 vs. 8.7 ± 5.7 scores)* p* > 0.5.

**Figure 4:**
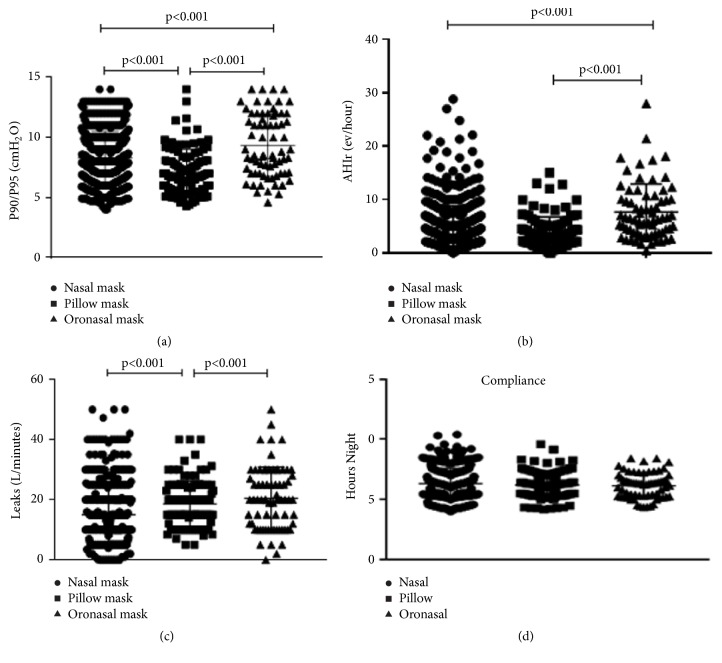
Comparison of effective pressure in the 90/95 percentile for APAP (a), residual apnea-hypopnea index (b), mean leak in L/m (c), and APAP adherence for different types of interfaces (d).

Our findings reveal differences in effective pressure levels of obtained titration according to the device (P90/P95) (GI: 7.13 ± 1.9 vs. GII: 8.3 ± 2.1 vs. GIII: 9.3 ± 2.6 cmH_2_O,* p *< 0.001) (Figure 4(a)). Pressure values titrated through visual analysis of pressure-time curves were similar for all study nights across groups (GI: 7.9 ± 1.4 vs. GII: 8.6 ± 1.6 vs. GIII: 9.2 ± 1.9 cm of H_2_O,* p* > 0.5).

Residual AHI was lower for nasal pillows (GI: 3.7 ± 2.9 vs. GII: 4.6 ± 4.1 vs. GIII: 7.6 ± 5.2 events/hour,* p *< 0.001) (Figure 4(b)).

Leaks were considered acceptable in all groups but significantly lower for nasal masks (GI: 18.6 ± 7.7 vs. GII: 15 ± 9.8 vs. GIII: 20.4 ± 10.4 liters/min,* p* < 0.001) (Figure 4(c)).

Finally, no differences were found in terms of CPAP use per night (in hours) among the different types of masks (GI: 6.3 ± 1.2 vs. GII: 6.2 ± 1.1 vs. GIII: 6.1 ± 1.0,* p* < 0.4) (Figure 4(d)).

## 4. Discussion

The main finding of this study revealed that nasal pillows were as effective as nasal masks at treating obstructive disorders with CPAP therapy and achieving adherence during titration in CPAP-naïve patients.

According to our data, CPAP-naïve patients can correctly use and adhere to auto-adjusting CPAP therapy during unmonitored titration after basic training and an interface-selection process.

Literature validates the use of auto-adjusting devices for titration purposes. This approach has several advantages: procedures are simpler, patients do not need an appointment with the sleep laboratory, and sleep laboratories are used only for cases where home-based titration is not effective or possible [16–22].

We conducted a visual analysis of pressure/time and leak/time curves to define final effective pressure. In statistical analysis, bench studies tend to show higher values for automatic algorithms because the software cannot exclude periods of improper mask fit [26]. In our study, visual analysis allowed us to identify and exclude pressure values related to those events. In addition, the rAHI value in this type of devices is reliable when < 10 events/hour is recorded, as compared to manual titration by PSG [27]. Our results, however, cannot be extrapolated to all available algorithms because of potential marked variations in the performance of each device. Our analysis shows that a careful interface selection and initial guidance enable most CPAP-naïve patients to use CPAP therapy > 4 hours/night and correct their AHI.

Some studies that assessed adherence after 3 months showed that APAP titration improves mean compliance in approximately one hour, when compared to PSG titration at the sleep laboratory [28–30]. In our study, mean compliance with auto-adjusting CPAP in CPAP-naïve patients was > 6 hours in a mean period of > 3 nights.

The influence of masks on CPAP working pressure has been previously described [13] as being higher for oronasal masks. This is consistent with our findings, although only a minority of patients in our series used this type of mask. Borel et al. [13] conducted a descriptive study based on 2.311 OSA patients and found statistically significant differences between the 3 groups of masks in terms of CPAP titration pressure, which was higher with oronasal masks and lower with nasal masks and nasal pillows. Conversely, adherence was better with smaller masks. We found lower pressures (P90/P95) in the groups of nasal masks and pillows. A remarkable finding was that oronasal masks were preferred by more severe cases, while nasal pillows were mostly chosen by patients with mild-moderate AHI.

Interfaces have proved essential in promoting adherence. Meslier et al. [31] carried out a survey among > 3.000 patients and found that half of them rated their interface as being “good” or “very good”, while the other half reported they were not satisfied with it [30]. Available data shows that a first good experience during the implementation of treatment (during the first week) is crucial for long-term adherence [31–34].

During the last years, some studies have shown that the performance of nasal pillows is similar to that of nasal masks. Therefore, nasal pillows could become first-line choice for CPAP therapy [8, 13, 15]. In 2011, Ryan et al. [15] reported that 62% of patients preferred nasal pillows over nasal masks. In our sample, nasal pillows and nasal masks showed equally lower leak rates, confirming prior observations.

A recent literature review analyzed 2 properly designed studies that compared the 3 types of masks in terms of CPAP therapy efficacy. Ebben et al. [12] assessed 55 OSA patients who were randomized to oronasal masks, nasal masks, or nasal pillows for CPAP titration. Nasal masks and nasal pillows were similar in terms of CPAP levels and consistently lower than oronasal masks. They found no differences in terms of residual AHI.

We found no difference in CPAP compliance with the 3 types of masks, following the titration protocol currently in force in our center.

## 5. Limitations

One of the limitations of our study is its retrospective design. In addition, a source of bias may derive from the lack of randomization to mask type. Still, our study is based on a series of consecutive patients that underwent the same clinical protocol.

The education session and practical demonstration of available interfaces seemed effective and allowed us to achieve acceptable adherence in CPAP-naïve patients. Besides, allowing patients to take an active part in the mask selection process may have exerted a positive influence in treatment adherence. Higher socioeconomic levels and older ages are associated with better compliance and may partially account for our findings, since we analyzed an adult population (≈ 60 years of age) referred from a private care setting for home-based titration (possible selection bias). On the other hand, clinical management by trained personnel and the influence of cognitive-behavioral strategies could also have improved short-term compliance [30, 35].

## 6. Conclusions

During home-based auto-adjusting CPAP titration by CPAP-naïve patients, nasal masks were selected by most participants and had lower leak rates. Nasal pillows presented a similar performance.

## Figures and Tables

**Table 1:** Population related variables.

Variable	Value
n	707

Men	513 (83%)

Age (years)	59.1 ± 12.2

BMI (kg/m^2^)	34.1 ± 6.7

Epworth Sleepiness Scale	9.77 ± 5.83

Diagnosis by PSG	212 (30%)

Diagnosis by RP	495 (70%)

Values expressed as means and standard deviation (±).

## Data Availability

The clinical information about patients and titration test used to support the findings of this study may be released upon application to the institutional revision committee of Hospital Británico de Buenos Aires, Argentina, who can be contacted at CRI@hbritanico.com.ar.
